# Anticancer Molecular Mechanism of Protocatechuic Acid Loaded on Folate Coated Functionalized Graphene Oxide Nanocomposite Delivery System in Human Hepatocellular Carcinoma

**DOI:** 10.3390/ma14040817

**Published:** 2021-02-09

**Authors:** Kalaivani Buskaran, Saifullah Bullo, Mohd Zobir Hussein, Mas Jaffri Masarudin, Mohamad Aris Mohd Moklas, Sharida Fakurazi

**Affiliations:** 1 Laboratory for Vaccine and Immunotherapeutic, Institute of Biosciences, Universiti Putra Malaysia, Serdang, Selangor 43400, Malaysia; vaneey_88@yahoo.com; 2 Materials Synthesis and Characterization Laboratory, Institute of Advanced Technology, Universiti Putra Malaysia, Serdang, Selangor 43400, Malaysia; bullosaif1@gmail.com (S.B.); mzobir@upm.edu.my (M.Z.H.); 3 Department of Cell and Molecular Biology, School of Biotechnology, Universiti Putra Malaysia, Serdang, Selangor 43400, Malaysia; masjaffri@upm.edu.my; 4 Department of Human Anatomy, Faculty of Medicine and Health Sciences, Universiti Putra Malaysia, Serdang, Selangor 43400, Malaysia; aris@upm.edu.my

**Keywords:** graphene oxide, protocatechuic acid, nanocomposite, drug delivery, folic acid targeting, anticancer mechanism, HepG2 cell, hepatocellular carcinoma

## Abstract

Liver cancer is listed as the fifth-ranked cancer, responsible for 9.1% of all cancer deaths globally due to its assertive nature and poor survival rate. To overcome this obstacle, efforts have been made to ensure effective cancer therapy via nanotechnology utilization. Recent studies have shown that functionalized graphene oxide (GO)-loaded protocatechuic acid has shown some anticancer activities in both passive and active targeting. The nanocomposites’ physicochemical characterizations were conducted. A lactate dehydrogenase experiment was conducted to estimate the severity of cell damage. Subsequently, a clonogenic assay was carried out to examine the colony-forming ability during long-term exposure of the nanocomposites. The Annexin V/ propidium iodide analysis showed that nanocomposites induced late apoptosis in HepG2 cells. Following the intervention of nanocomposites, cell cycle arrest was ascertained at G2/M phase. There was depolarization of mitochondrial membrane potential and an upregulation of reactive oxygen species when HepG2 cells were induced by nanocomposites. Finally, the proteomic profiling array and quantitative reverse transcription polymerase chain reaction revealed the expression of pro-apoptotic and anti-apoptotic proteins induced by graphene oxide conjugated PEG loaded with protocatechuic acid drug folic acid coated nanocomposite (GOP–PCA–FA) in HepG2 cells. In conclusion, GOP–PCA–FA nanocomposites treated HepG2 cells exhibited significant anticancer activities with less toxicity compared to pristine protocatechuic acid and GOP–PCA nanocomposites, due to the utilization of a folic acid-targeting nanodrug delivery system.

## 1. Introduction

Liver cancer is recorded as the fifth prevailing cancer, which is responsible for 9.1% of deaths from all cancers globally [1]. Due to its assertive nature and poor survival rate, it still remains an important public health concern worldwide [2]. Hepatocellular carcinoma (HCC) or hepatoma is known as a primary malignant neoplasm derived from hepatocytes, accounting for about 75–90% of all liver cancers [1]. The American Cancer Society estimated there were about 40,710 cases, with 29,200 (men) and 11,510 (women) new cases of HCC, and intrahepatic bile duct cancer was present in about 28,920 cases, with 19,610 (men) and 9310 (women) deaths due to liver cancer [3]. The epidemiology of HCC showed the regions with the highest incidences to be Asia–Pacific (East Asia and Southeast Asia), and Central and Western Africa, wherein about 85% of the cases were identified [4]. 

Protocatechuic acid (3, 4-dihydroxybenzoic acid; PCA) [5] is a phenolic compound which isolated from plants, fruits, nuts, green tea and black rice [6], and is well-known for its properties of inducing caspase-mediated apoptosis activity and enhancing the cytotoxicity effect on various cancer cell lines. These phenolic compounds are mainly responsible for the apoptotic, radical-scavenging, antioxidant, and pro-oxidant characteristics of the antitumor activities [7]. This natural product possesses good therapeutic potential, and low toxicity and side effects. Despite the advantages concerning the biocompatibility, the poor bioavailability, solubility and absorption of natural compounds presents a greater challenge to using them as medicine [8].

Accordingly, a nanodelivery system that specifically delivers nanomedicine has emerged as a prominently developing sector in the field of medical science in this era [9]. This technologic expansion in nanomedicine has successfully branched out to ground advancements in drug manufacturing, drug delivery system and medical diagnostics [10]. Moreover, these developmental efforts have been made to overcome obstacles such as biocompatibility, bioavailability, solubility and non-specific delivery in cancer therapy, particularly enhancing the efficacy in treatment strategy. Nanotechnology has been a game-changer for advanced medicine, drug formulation, diagnostics, and targeting drug release and delivery [11].

Graphene oxide (GO) is known as a favorable functionalized nanomaterial that is being vastly utilized in drug delivery, photocatalysis, biosensing, electronics, agriculture, aquaculture, and also in energy loading devices such as super capacitors and batteries [11,12]. This two-dimensional nanocomposite with a single carbon layer has attracted enormous attention for its relevance in anticancer drug loading and delivery [13]. Moreover, folic acid (FA) is used to target the folate receptors. These folate receptors are abundantly expressed in cancer cells compared to healthy cells [14]. These cell surface glycoproteins are able to bind to the high-affinity folic acid and mediate a unidirectional transport of folate into cells. As such, the formulation of the targeted drug delivery system with anticancer drugs will maximize anticancer efficacy by minimizing side effects in healthy tissues [15].

In our previous study, we reported that a polyethylene glycol-conjugated graphene oxide (GOP) nanocomposite loaded with protocatechuic acid in both passive and active forms caused a reduced cell proliferation rate in HepG2 cell lines [16]. In addition, a preliminary study on the morphological changes in HepG2 cells treated with GOP–PCA and GOP–PCA–FA nanocomposites indicated that the mode of cell death mechanism in human hepatocellular carcinoma cells is apoptosis [17]. Intrigued by this result, a further investigation was conducted to explore the anticancer molecular mechanism to substantially validate the apoptotic cell death mechanism. This in vitro anticancer mechanism of the nanodrug delivery system is important for the better understanding of cellular toxicity and to forecast the potential anticancer molecular mechanism.

## 2. Materials and Methods

### 2.1. Materials

Graphite flakes (109 meshes), sodium dodecyl sulfate, sulphuric acid (H_2_SO_4_ 98%), phosphoric acid (H_3_PO_4_), potassium permanganate (KMnO_4_) and hydrogen peroxide were from Sigma Aldrich (St Louis, MO, USA) and were used without further purification. Hydrochloric acid (HCl, 37%), Diethyl ether, ethyl alcohol (99.7% *v*/*v*), sodium hydroxide, acetic acid glacial and 37% formaldehyde were from Friedemann Schmidt (Parkwood, WA, USA). Pristine protocatechuic acid (PCA) compound was purchased from Sigma Aldrich, USA. We also used the following: LDH test-kit (CytoTox 96^®^ Non-Radioactive Cytotoxicity Assay, Promega Co., Madison, WI, USA), Annexin V/FITC Apoptosis Detection Kit I (BD Biosciences, San Jose CA, USA), Propidium iodide (PI) Sigma-Aldrich, (St. Louis, MO, USA), CycleTESTTM PLUS DNA Reagent Kit (BD Biosciences, San Jose, CA, USA), JC-1 Mitochondrial Membrane Potential Assay Kit (Abnova, Middlesex County, NJ, USA), OxiSelect™ IntracellularROS Assay Kit (Green Fluorescence) (Cell BioLabs, Inc., San Diego, CA, USA). Phosphate Buffer Solution, methanol, and ethanol were all sourced from Sigma Aldrich (St. Louis, MO, USA).

### 2.2. Culture Cell Line 

HepG2 (HEPG2) (ATCC^®^ HB-8065™) cells were purchased from ATCC (Manassas, VA, USA). The cells were maintained in Dulbecco’s Modified Eagle Media (DMEM) supplemented with 10% *v*/*v* Fetal Bovine Serum (FBS), 1% *v*/*v* penicillin (100 units/mL) and streptomycin (100 μg/mL). The cells were cultured at 37 °C in a humidified 5% CO2 incubator. After 24 h, when the cell reached 80–90% confluency, they were trypsinized and seeded in a 96-well plate for further experiments.

### 2.3. Synthesis of Graphene Oxide (GO)

Graphite powder (3 g) was diluted into a mixture of concentrated sulfuric acid (H_2_SO_4_) (360 mL) and concentrated H_3_PO_4_ (40 mL). Next, 18 g KMnO_4_ was added into the concoction. The solution was stirred until it reached 50 °C for 12 h. Later, the suspension received 3 mL of hydrogen peroxide and the temperature of the resultant was reduced to 35 °C by adding ice cubes to the mixture. The color of the solution was changed to a yellow suspension. The resultant GO was separated by a filtration process. The remaining solid was washed with 200 mL HCl three times. The solution was then centrifuged at 8000× *g* rpm for 10 min. The final mixture was washed with deionized water three times to purify the solid mixture. The sample was sonicated for 30 min at 40 kHz. Finally, the sample was dried at 40 °C for between 24 and 48 h to obtain dry graphene oxide flakes [16].

### 2.4. Conjugation of Graphene Oxide with Polyethylene Glycol (GOP) Nanocarrier System

In order to conjugate PEG to GO, an esterification reaction was conducted between the carboxylic acid group from GO and the hydroxyl group from PEG. The GO suspension (2 mg/mL) was mixed in 20 mL of sodium hydroxide. The mixture was then sonicated. The pH of the solution was titrated by adding 3 mL hydrochloric acid (HCl) to obtain pH 5. The sample was centrifuged at 8000× *g* rpm, 25 °C for 15 min to produce GO carboxylic acid (GO-COOH). The activated carboxylic acid group in GO was catalyzed using 400 mg *N*-(3-Dimethylaminopropyl-*N*’-ethylcarbodiimide hydrochloride (EDC) and 240 mg *N*-hydroxysulfosuccinimide (NHS) and stirred for 24 h. Next, 1.5 g of PEG4000 was added to the above suspension and constantly stirred overnight. Finally, the suspension was washed with deionized water and centrifuged at 8000× *g* rpm, 25 °C for 30 min. Then suspension (GOP) was dried at 40 °C for between 24 and 48 h to obtain dry graphene oxide conjugated to PEG [16].

### 2.5. Protocatechuic Acid Loaded on GOP and Coated with Folic Acid (FA)

For protocatechuic acid (PCA) drug loading, 5 g of PCA was loaded into 100 mL of the GOP nanocarrier solution, and the sample was stirred for 24 h. Later, the sample was centrifuged at 8000× *g* rpm, 25 °C for 15 min. This was followed by washing thoroughly with deionized water and drying at 40 °C. This resulted in the synthesis of graphene oxide with polyethylene glycol loaded with protocatechuic acid (GOP–PCA). The flaky material was then grounded into powder and resuspended in 50 mL of 1% folic acid solution and stirred for 24 h. The sample was then washed thoroughly with deionized water and dried in the oven at 40 °C. This gave conjugated GO with PEG, which was loaded with protocatechuic acid and finally coated with folic acid nanocomposite (GOP–PCA–FA), and then grounded to fine powder and subjected to further physicochemical characterization [16].

### 2.6. Physicochemical Characterization of Nanocomposites

The X-ray diffraction pattern was used to analyze the crystalline phase of the PCA drug, folic acid, GO, GOP, the GOP–PCA nanocomposite and the GOP–PCA–FA nanocomposite. The patterns were recorded using CuKα radiation (λ = 1.5418 Å) at 30 kV and 30 mA of an XRD-6000 Diffractometer (Shimadzu, Tokyo, Japan). The particle size distribution and zeta potential of the GOP–PCA nanocomposite and the GOP–PCA–FA nanocomposite were measured at room temperature by dynamic light scattering (DLS nanosizer, MALVERN, NanoS, Worcestershire, UK). Field Emission Scanning Electron Microscope (FESEM) images of the samples were recorded using a field emission scanning electron microscope, Nova NanoSEM 230 model (Denton, TX, USA). All the samples used for this analysis were in powder form. Fourier-transform infrared spectroscopy (FTIR) by PerkinElmer FT-IR spectrometer (SPECTRUM 1000) (Ynysmaerdy, UK) shown in Figure S1 and Table S1, Transmission electron microscope (TEM, Hitachi H-7100, Tokyo, Japan) micrographs shown in Figure S2.

### 2.7. Determination of Encapsulation Efficacy and Loading Content Using HPLC Analysis

The drug encapsulation efficacy and loading content of the PCA percentage in the nanocomposites were quantified using a Waters HPLC model 2695 equipped with an Agilent C18 column, photodiode array (PDA) detector and Empower software (Waters, Milford, MA, USA). Stock solutions of nanocomposites were made in methanol with concentrations of 0.5 mg/mL. The range of working standard solutions for nanocomposite concentrations (10–300 µg/mL in acetyl nitrate) was prepared by diluting the stock solution in pure methanol. The samples with a volume of 20 µL were injected into the column and detected at 210 nm. Data were collected and standard calibration curves were plotted to determine the unknown nanocomposite concentration. The encapsulation efficiency (%EE) and the loading content (%LC) of GOP–PCA and GOP–PCA–FA nanocomposites were calculated using the following formula:

EE (%) = (Total nanocomposite with PCA − Free PCA)/(Total nanocomposites with PCA) × 100(1)

LC (%) = [The weight of PCA in nanocomposites/the weight of nanocomposites] × 100(2)

### 2.8. Protocatechuic Acid In Vitro Drug Release from Nanocomposites

The in vitro PCA drug release from the nanocomposites was studied using a Perkin Elmer UV/Vis spectrophotometer (Model Lambda 35, Mundelein, IL, USA). The lambda max for the PCA (256 nm) wavelength was used to perform the in vitro drug release studies. For these studies approximately 3 mg of PCA compound and nanocomposites were added into 30 mL of pH 7.4 (blood) and pH 4.8 (intracellular lysosomal pH) buffers separately, and the release profiles were determined by taking the absorption percentage at different time intervals for 144 h.

### 2.9. Lactate Dehydrogenase Assay for Plasma Membrane Integrity Analysis

The HepG2 cells were grown on a 96-well plate with a seeding density of 1 × 10^4^ cells/well for overnight. Once they reached confluence, the cells were treated with GOP nanocarrier, pristine PCA compound, and GOP–PCA and GOP–PCA–FA nanocomposites at different concentrations of 1.56–100 µg/mL and incubated for another 72 h. For the positive control, 10 µL of lysis buffer solution was added to the untreated (control) cells 45 min before taking the absorbance reading. From the treated cells, 50 µL of supernatant solution was pipetted onto a new 96-well plate as per instructions from the LDH assay kit. Next, 50 µL of detection reagents were added and incubated for 30 min away from light at room temperature. Finally, we added 50 µL of the stop solution, and the absorbance value for the lactate dehydrogenase was recorded at 490 nm on a Microplate Reader. The release percentage of LDH expression was calculated using the following calculation:

Percentage of LDH release = Experimental LDH release (OD490)/Maximum LDH release (OD490) × 100(3)

where in Experimental LDH release is from the cells treated with nanocomposites, and Maximum LDH release is the positive control for the LDH of the cells.

### 2.10. Colony Formation Evaluated by Clonogenic Assay 

This assay was conducted to obtain the colony-forming ability of the HepG2 cells when treated with GOP nanocarrier, pristine PCA, GOP–PCA and GOP–PCA–FA nanocomposites. The HepG2 human hepatocellular carcinoma cells were plated individually into 100 mm petri dishes until cell densities of 200 cells/dish were obtained in 24 h. Once the cells were counted, they were treated with the pristine PCA drug, nanocarrier and nanocomposites at concentrations of 38 µg/mL each for 72 h. After the 72 h treatment, the culture media was replaced with fresh 5 mL DMEM media. This medium was changed frequently at 2-day intervals for the next 10 days. On the final day of viewing for colony formation, the cells were fixed with cold methanol and acetic acid for 5 min. The fixed solution was carefully aspirated and washed with cold PBS. Finally, the cells were stained with 0.5% (*w*/*v*) crystal violet for 30 min and washed with distilled water three times. The dishes were air dried before the colony formation was counted under a light microscope. The colonies of 50 cells clumped together were calculated manually using a microscope and standardized with untreated (control) cells. The survival fraction (SF) of colony formation was calculated using the following method:

Survival Fraction (SF) = PE of treated colonies/PE of untreated (control) × 100(4)

where PE stands for plating efficiency, which is calculated using

Plating Efficiency (PE) = Total number of colony counted/Total number of colony plated × 100(5)

### 2.11. Apoptosis/Necrosis Cell Death Analysis 

The cells were grown in a 25 cm^2^ culture flask until a density of 1 × 10^6^ cells/mL was reached. Then, the cells were treated with the pristine PCA drug, GOP nanocarrier, GOP–PCA nanocomposite and GOP–PCA–FA nanocomposite at 38 µg/mL concentrations (IC_50_ of PCA) (Figure S3 and Table S2) [16] for 72 h. As a negative control, untreated (control) cells were cultured in DMEM medium alone. Once the cells were treated for 72 h, the cultured medium was discarded and washed with PBS. Then, the cells were harvested by trypsinization, washed with cold PBS and centrifuged to obtain a cell pellet. Finally, cell culture solutions of 1 × 10^5^ cells/mL in 100 µL volume were counted and transferred into 5 mL flow tubes for each experiment. The cell solution was mixed together with 5 µL of Annexin-V/FITC and 5 µL of PI and gently vortexed by avoiding the formation of air bubbles. Solutions were incubated for 15 min at room temperature in the dark. As the last step, 400 µL of 1× Binding buffer was added into each tube, and the samples were analyzed using a flow cytometer (BD FACS Canto II, San Jose, CA, USA). The flow cytometer was programmed to read 10,000 events for each set of experiments, which then further separated them into four different quadrants of heterogeneous populations. Each quadrant was pooled into the sub-populations of unstained cells known as viable cell and cells that were stained with Annexin-V/FITC only, labeled as early apoptotic cells, whereas cells stained with both the Annexin-V/FITC and PI were categorized as late apoptotic cells, and lastly cells stained with PI only were known as necrotic cells.

### 2.12. Cell Cycle Arrest Analysis Using Propidium Iodide 

Propidium iodide (PI) staining was used to determine the cell cycle arrest of HepG2 cells after treatment. The HepG2 cells were cultured in a 25 cm^2^ culture flask until the cells reached a density of 1 × 10^6^ cells/mL. Cells were treated with pristine the PCA drug, GOP nanocarrier, GOP–PCA nanocomposite and GOP–PCA–FA nanocomposite at 38 µg/mL concentrations each for 72 h. As a negative control, untreated (control) cells were cultured in DMEM medium alone. Once the cells were treated for 72 h, cultured medium was discarded and washed with PBS. Then the cells were harvested using a scrapper, washed with cold PBS and centrifuged to obtain a cell pellet. The final cell densities for each experiment of 5 × 10^5^ cells were counted and transferred into 5 mL flow tubes. Cells were resuspended with 250 µL solution A (trypsin Buffer) and 250 µL solution B (trypsin inhibitor and RNAse buffer), and finally 200 µL of PI staining solution was added and incubated on ice for 10 min. The samples were analyzed in a flow cytometer. The flow cytometer was programmed to read 10,000 events for each set of experiments. The linearity of the flow cytometer was tested using a BD DNA QC Particles kit for more accurate results. The cell cycle arrest results were presented in the sub G0/G1, G0/G1, S and G2/M phases, and analyzed using Modfit software Version 3.2.

### 2.13. Mitochondrial Membrane Potential Analysis

Mitochondrial membrane potential analyses of HepG2 cells were performed using a JC-1 fluorescence staining assay. The HepG2 cells were grown in a 25 cm^2^ culture flask until the cells reached a density of 1 × 10^6^ cells/mL. Then, the cells were treated with pristine PCA drug, GOP nanocarrier, GOP–PCA nanocomposite and GOP–PCA–FA nanocomposite at 38 µg/mL concentrations each for 72 h. As a negative control for this experiment, untreated (control) cells were cultured in DMEM medium alone, whereas positive control cells were incubated with 10 µM of CCCP solution. Once the cells were treated for 72 h, cultured medium was discarded and washed with PBS. Then the cells were harvested using a scrapper, washed with cold PBS and centrifuged to obtain cell pellets. Final cell densities of 5 × 10^5^ were counted and transferred to a flow tube, then resuspended with 100 µL/mL of JC-1 dye and incubated in 5% CO_2_ incubator for 15 min. After the incubation, the cells were centrifuged at 400 × g for 5 min. Finally, the sample was resuspended in 1 mL JC-1 Assay Buffer and analyzed in a flow cytometer (BD FACS Canto II). The flow cytometer was programmed to read 10,000 events for each set of experiments.

### 2.14. Measurement of Cellular Reactive Oxygen Species

The HepG2 cells were seeded in a 96-well plate and grown for 24 h until the cells reached a density of 1 × 10^4^ cells/well. Cells were treated with pristine PCA drug, GOP nanocarrier, GOP–PCA nanocomposite and GOP–PCA–FA nanocomposite at 38 µg/mL concentrations each for 72 h. For the negative control, untreated (control) cells were culture in DMEM medium alone, whereas the positive control cells were incubated with 200 mM H_2_O_2_ for 1 h before adding the DCFH-DA probe. Once the cells were treated, the culture medium was replaced with 100 µL of 20 µM oxidant-sensitive dye DCFH-DA and incubated for 1 h at 37 °C. The cells were washed with D-Hanks buffer solutions twice after 1 h incubations. Finally, 100 µL of D-Hanks buffer solution was added into each well, and the fluorescence intensity was recorded by a Microplate Reader at the excitation and emission wavelengths of 435 nm and 585 nm, respectively. The ROS fluorescence intensity ratio was calculated using the formula:

ROS intensity ratio = (F test − F blank)/(F control − F blank)(6)

where F test stands for the fluorescence intensity of the treated cell or positive control, F control is the fluorescence intensity of untreated cells and F blank is the fluorescence intensity of empty wells without cells.

### 2.15. Proteome Profiler Human Apoptotic Antibody Array Detection

The apoptotic proteins were induced when the HepG2 cells were treated with nanocomposite and evaluated by Human Apoptosis Antibody Array kit (Ray Biotech Inc., Peachtree Corners, GA, USA). The HepG2 cells were treated with 18.89 µg/mL of nanocomposite for 72 h. As a negative control, untreated (control) cells were culture in DMEM medium alone. A total of 1 × 10^7^ cells/treatment cells were resuspended in lysis buffer (500 µL) for 30 min and vortexed until the cells lysed completely. Then, the lysed cells were centrifuged for 30 min at 14,000 × *g* at 4 °C. The remaining supernatants were discarded. The array membrane was carefully removed from protective sheets and placed on the array buffer 1 for 1 h, which served as a blocking buffer to block the unspecific binding sites. Once incubated, array buffer 1 was removed and replaced with 250 μL/array of lysate protein with 400 μg/mL total protein concentration and diluted in 1.25 mL of array buffer 1. Diluted protein samples were smeared on the array membrane and were allowed to incubate overnight with gentle rocking at 4 °C.

The array membrane was then washed 3 times with 1× wash buffer for 10 min on a rocking platform. Consecutively, 15 μL of reconstituted Detection Antibody Cocktail was diluted in 1.5 mL 1× array buffer 2/3 and applied on the array membrane. Incubation was continued for 1 h at room temperature (25 ± 2 °C). Membranes were washed; streptavidin-(HRP) was added and incubated for 30 min to enhanced the chemiluminescence detection. The membranes were washed thoroughly and proceeded for detection. Blots were detected via ChemidocTM XRS system (Bio-Rad, Hercules, CA, USA). Protein loading densities were compared to the control antibodies to measure relative densities. 

### 2.16. Apoptotic Gene Expression Markers Detection

Total RNA was obtained from cells by using FavorPrep total RNA mini kit (Favorogen Biotech, Taiwan) following the instructions sheet. The master mix for RT-PCR was formulated according to Table 1. The template RNA mixture was added to the master mixture and pipetted slowly. Following this, the prepared master mixtures were aliquotted into PCR reaction tubes. All the samples were preheated at 95 °C for 5 min to break the RNA double strands. The tubes were incubated on ice for 5 min before starting to arrange the samples into the thermocycler. Each RT reaction consists of 7 μL master mix, 3 μL primer, and 5 μL cDNA sample. The samples were run for 40 cycles with the following parameters; temperature of 16 °C for 30 s, 42 °C for 30 s and 45 °C for 30 s. The results were analyzed using sequence detection system software (BioRad CFX 96 PCR Detection System, Hercules, CA, USA).

The quantitative real time polymerase chain reaction (qRT-PCR) was conducted by preparing the total components of the PCR reaction mixture according to Table 2. Once the sample proteins were mixed gently, the PCR tubes were centrifuged briefly to eliminate entrapped air bubbles and spin the content to the bottoms of the tubes. Next, the PCR strip tubes were placed in an Applied Bio systems 7900 HT Fast Real-Time PCR holder (Mundelein, IL, USA) and we initiated the programmed 40 cycles with a denature temperature of 95 °C for 15 s, and an annealing and extending temperature of 60 °C for 60 s. The Ct values of the gene of interest were normalized using the housekeeping gene (GAPDH). Finally, the gene expression level of the apoptotic proteins was standardized to a calibrator, and the GAPDH and fold change were determined using 2^−ΔΔCt. Table 3 shows the primer that was used.

### 2.17. Statistical Analysis

The quantitative data obtained from each assay were expressed as mean ± standard deviation (SD) from the three least independent experiments (n = 3 for each experiment). A normality test was performed using the Shapiro–Wilk test, whereas Levene’s test was used for the homogeneity test of variance. Statistical analysis was conducted using Analysis of Variance (ANOVA) followed by a Games–Howell post hoc test to consider the significant difference by using SPSS program Version 22. Two variable data were analyzed using unpaired *t*-test. When the *p* value < 0.05 it is considered to be statistically significant.

## 3. Results

### 3.1. X-Ray Diffraction Analysis

**Figure 1:**
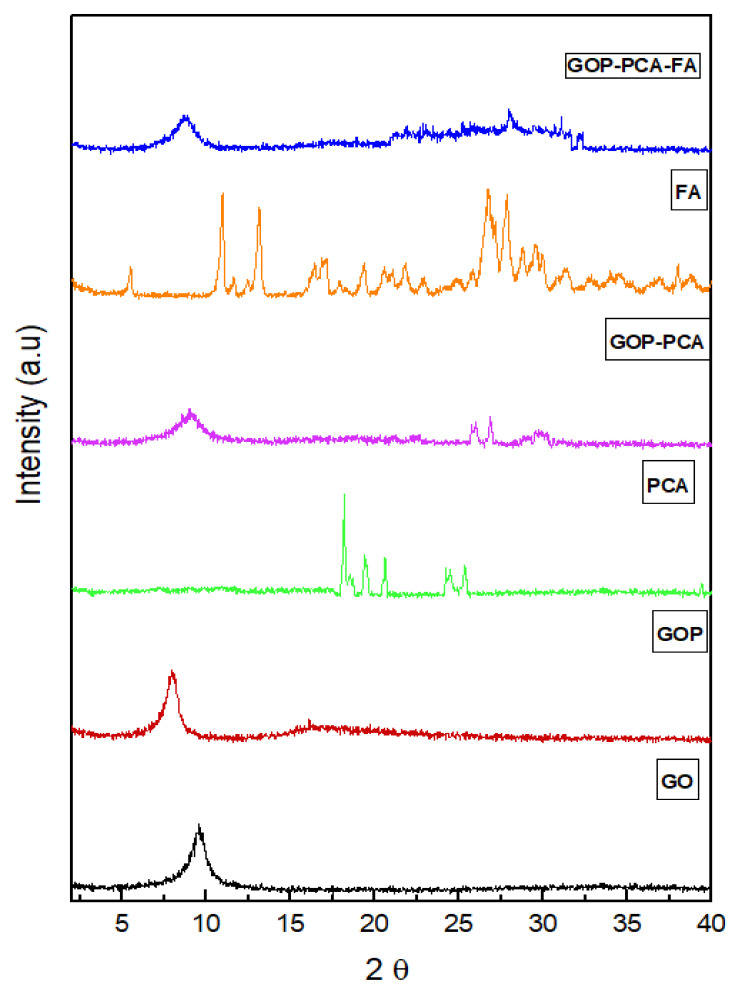
X-ray diffraction (XRD) peaks of graphene oxide (GO), graphene oxide-conjugated polyethylene glycol (GOP), pristine protocatechuic acid (PCA), GOP–PCA nanocomposite, folic acid (FA) and GOP–PCA–FA nanocomposite.

The X-ray diffraction in Figure 1 shows reflections of graphene oxide (GO), graphene oxide-conjugated polyethylene gycol (GOP) nanocarrier, pristine PCA, GOP–PCA nanocomposite, and the FA and GOP–PCA–FA nanocomposite formulation. The graphene oxide shows peaks at 2ϴ = 9.72° with basal spacing of 8.71 Å, while the nanocarrier (GOP) spectrum showed a strong peak at 7° and a small hub between 2ϴ of 15 and 25°, which may be due to the PEGylation process [18]. The pristine PCA has sharp reflections at 2θ = 18.1 and a small peak around 26.2, which matched the reflection patterns of pristine PCA [6]. The intensities of the reflections also increased when the GOP–PCA nanocomposite formed. There are few reflections that can be observed indicating the crystalline nature of the FA. The GOP–PCA–FA nanocomposite exhibits a peak broader than GO, and the spike was not as sharp as GOP with a few smaller peaks around 22–35°, which may indicate the formation of PCA coated FA was successful. The increase in intensity indicated the increase in the crystallinity of the nanocomposite.

### 3.2. Determination of Size Distribution and Zeta Potential Measurement of Nanocomposites

**Figure 2:**
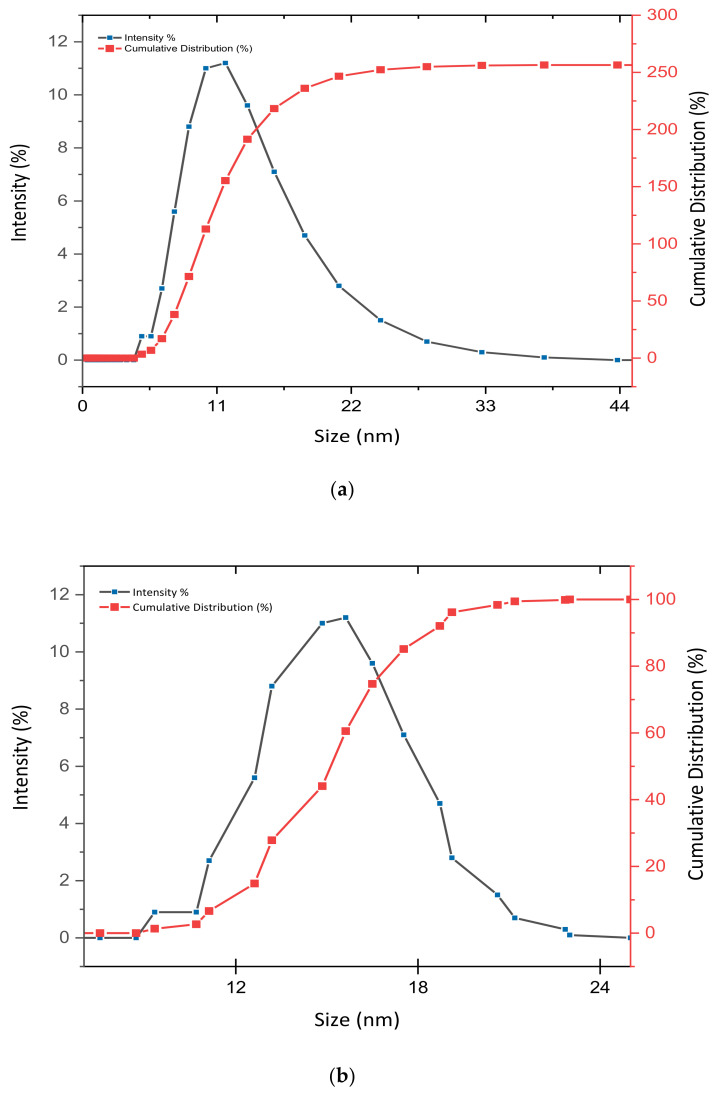
Particle size, relative and cumulative distributions of the (**a**) GOP–PCA nanocomposite and (**b**) GOP–PCA–FA nanocomposite.

Dynamic light scattering (DLS) analyses were applied to determine the particle size and zeta potential measurement of the nanocomposites in aqueous solution. Figure 2a (GOP–PCA) and Figure 2b (GOP–PCA–FA) show the particle size distribution and cumulative distribution of nanocomposites. The GOP–PCA nanocomposite encompasses average sizes of 14 ± 1.53 nm with a narrow distribution of particle sizes between 5 and 25 nm, while the size of the GOP–PCA–FA nanocomposite was found to be between 10 and 25 nm with an average size of 17 ± 2.08 nm. Table 4 shows the zeta potential value of the nanocarriers and nanocomposites.

### 3.3. Surface Properties Analysis

**Figure 3:**
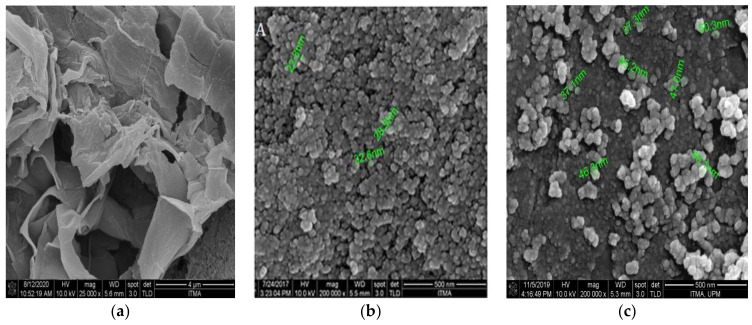
FESEM image of the (**a**) GO (**b**) GOP–PCA nanocomposite and (**c**) GOP–PCA–FA nanocomposite.

The surface and morphology of the graphene oxide nanocomposites were observed using a field emission scanning electron microscope (FESEM). Figure 3a depicts the surface morphology of graphene oxide alone, wherein the sheet structure of GO can be observed clearly. On the other hand, micrographs of Figure 3b (GOP–PCA) provide information on the interfacial interactions of the GOP-loaded pristine PCA drug entrapped on the nanocomposite. The surface of the sample is displayed in self-aggregated structures with an average size around 20–30 nm, which is due to the drug encapsulation and aggregations. Figure 3c (GOP–PCA–FA) represents GOP loaded with PCA and folic acid-coated nanocomposites, with an average size of 35–45 nm, which is relatively bigger due to the presence of FA as a coating agent. Overall, the synthesized nanocomposites show a spherical shape, a relatively narrow size distribution and relatively smooth surface. The nanocomposites barely show any isolated form of graphene oxide sheet, indicating the successful polymerization of graphene oxide with PEG and the entrapment of the drug molecules and folic acid.

### 3.4. Quantification of Encapsulation Efficacy and Drug Loading Analysis

High-Performance Liquid Chromatography (HPLC) was used to quantify the drug encapsulation/loading percentage in nanocomposites. In brief, PCA was prepared separately using five standards (10, 20, 30, 40, 50, 100, 150, 200 and 250 ppm). They were assessed using a mobile phase of acetonitrile: water in a ratio of 1:1 *v*/*v*, with adjusted pH 3. The R^2^ value of the calibration curve was found to be 0.9884. Table 5 displays the PCA percentage of encapsulation/loading in the GOP–PCA and GOP–PCA–FA nanocomposites, respectively.

### 3.5. Protocatechuic Acid in vitro Drug Release Study of Nanocomposite

**Figure 4:**
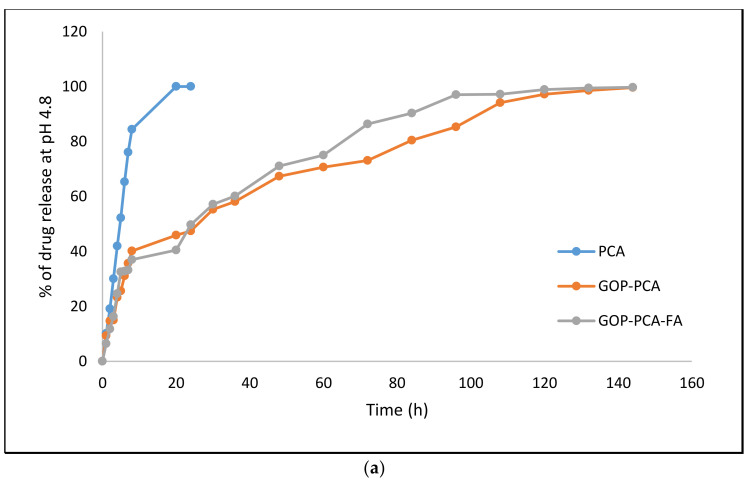

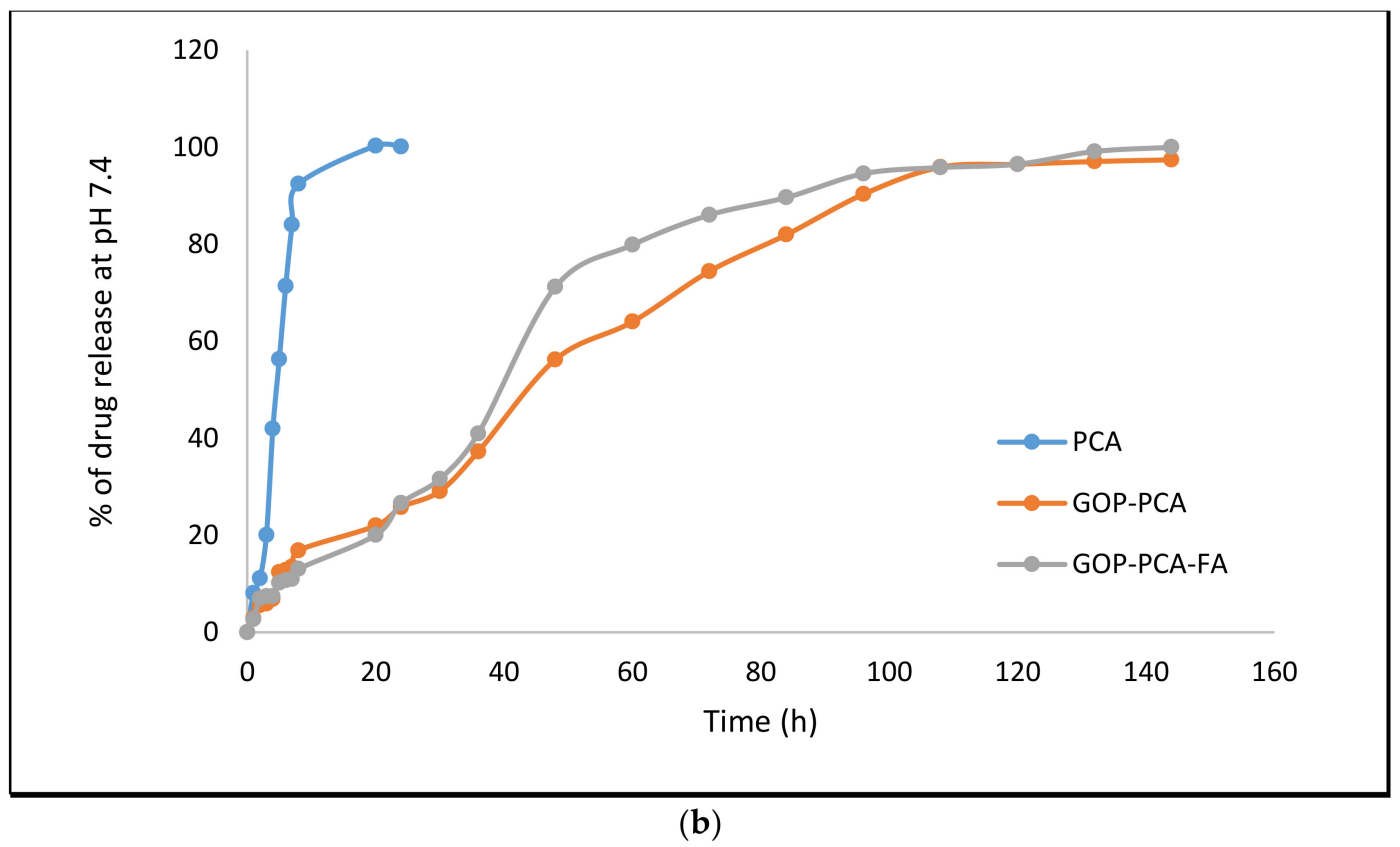
(**a**) In vitro release of PCA, GOP–PCA and GOP–PCA–FA in pH 4.8 solution., (**b**) in vitro release of PCA, GOP–PCA and GOP–PCA–FA in pH 7.4 solution.

Figure 4a,b show the in vitro drug release of the PCA drug alone and PCA from GOP–PCA and GOP–PCA–FA. This was conducted in a human body replicate situation, which is a phosphate buffer saline (PBS) solution of pH 7.4 (human blood pH) and pH 4.8 (intercellular lysosomal pH) at 37 °C with continuous shaking. The UV–Vis absorption spectrum for PCA (256 nm) was recorded as the individual lambda maxes. The pristine PCA drug showed complete release within the 24 h timeframe at both pH levels. PCA from the nanocomposite showed a burst released at pH 4.8 for the first 8 h compared to pH 7.4. After that, the release showed a more controlled sustained release. The maximum percentage of PCA drug released from GOP–PCA–FA at pH 4.8 was about 86%, compared to GOP–PCA (80%), at 72 h. The complete release of PCA from nanocomposites took about 144 h.

### 3.6. Lactate Dehydrogenase (LDH) Release from HepG2 Cells Treated with PCA Drug, GOP–PCA, and GOP–PCA–FA Nanocomposites

**Figure 5:**
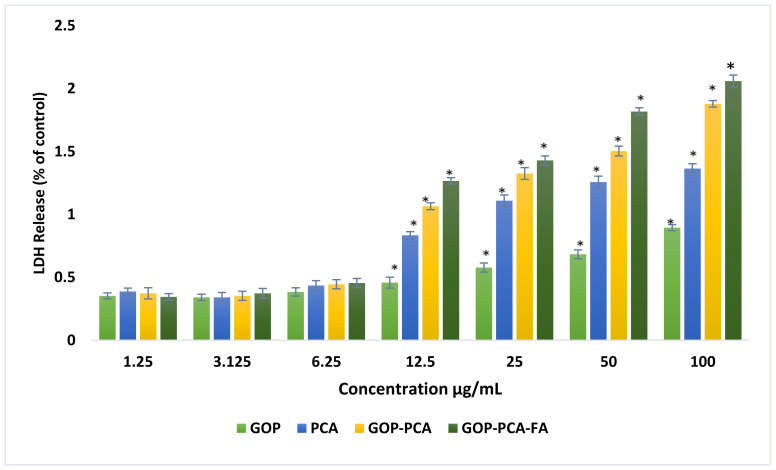
GOP nanocarrier, PCA drug, GOP–PCA and GOP–PCA–FA nanocomposite enhance lactate dehydrogenase release in HepG2 cells. All data are reported with mean ± SD of three independent experiments. The significant differences (*p* < 0.05) * were determined among GOP against PCA, GOP–PCA and GOP–PCA–FA by one-way ANOVA followed by Games–Howell post hoc tests.

As shown in Figure 5, the experiment was conducted using a concentration ranging from 1.25 to 100 μg/mL on HepG2 cells. The results proved that the influence of the cell membrane integrity of HepG2 cells responded in a dose-dependent manner to concentration. All the cells were treated with GOP nanocarrier, pristine PCA, GOP–PCA nanocomposite and GOP–PCA–FA nanocomposite at 12.5–100 μg/mL; there have been significant increases in the level of LDH released, as compared to the untreated cells. Cells treated with 1.25–6.25 μg/mL showed no significant difference in the LDH release level.

### 3.7. The Colony-Forming Ability of HepG2 Cells Following Long-Term Exposure to Nanocomposite

**Figure 6:**
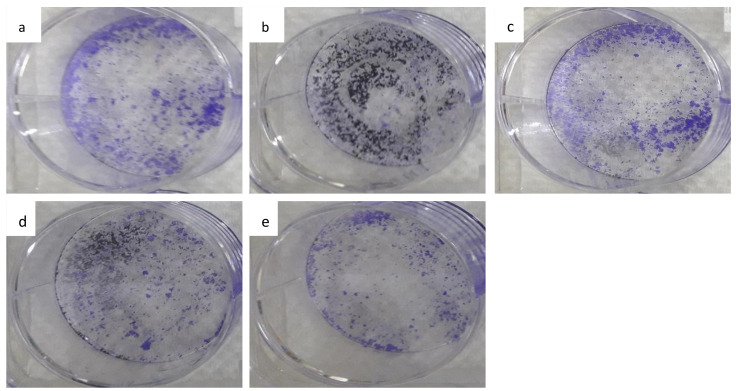
Colony-forming abilities of HepG2 cells were investigated by clonogenic assay with (**a**) untreated cells, (**b**) GOP, (**c**) PCA, (**d**) GOP–PCA and (**e**) GOP–PCA–FA nanocomposites.

**Figure 7:**
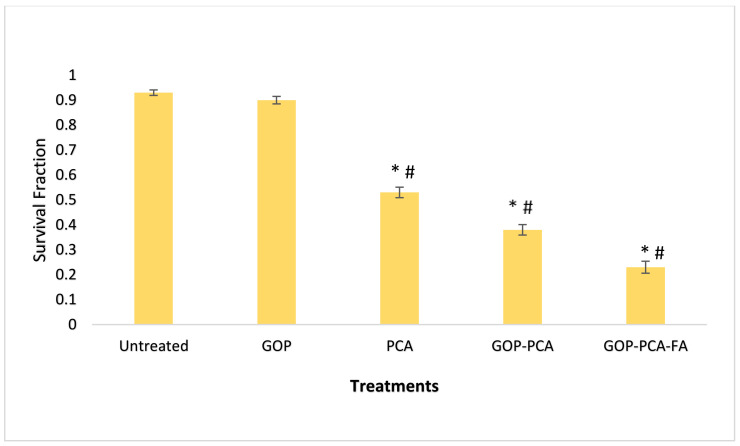
Clonogenic assay shows the long-term effects of the treatment of HepG2 cells with untreated, GOP nanocarrier, PCA drug, GOP–PCA nanocomposite and GOP–PCA–FA nanocomposite. Cells treated with GOP, PCA drug, GOP–PCA and GOP–PCA–FA nanocomposites at 38 μg/mL concentrations for 72 h. The significant differences (*p* < 0.05) * were determined among untreated HepG2 cells against GOP, PCA, GOP–PCA and GOP–PCA–FA, and (*p* < 0.05) # using GOP treated against nanocomposites using one-way ANOVA followed by Games–Howell post hoc tests.

The ability of HepG2 cells to form colonies and proliferate is shown in Figure 6; following treatment with a GOP nanocarrier, PCA, GOP–PCA, and GOP–PCA–FA, the nanocomposites were depicted. Figure 7 shows the survival fraction of the colony-forming ability of HepG2 cells wherein distinct reductions in PCA, GOP–PCA, and GOP–PCA–FA nanocomposites were observed compared to untreated cells. The untreated cells and GOP nanocarrier incubated cells did not show any significant effects on the colony-forming ability of HepG2 cells; hence a high survival fraction of the cells was observed. However, the untreated cells, as against the pristine PCA and nanocomposite-treated cells, showed significant (*p* < 0.05) reductions in colony formation. Subsequently, significant (*p* < 0.05) reductions were also observed in pristine protocatechuic acid and nanocomposite-treated cells compared to nanocarrier after 14 days. This proves the effectiveness of the nanocomposites in treating targeted cancer cells.

### 3.8. Determination of Nanocomposite-Induced Apoptosis in HepG2 Cells Using Annexin V FITC/Propidium Iodide (PI) Staining

**Figure 8:**
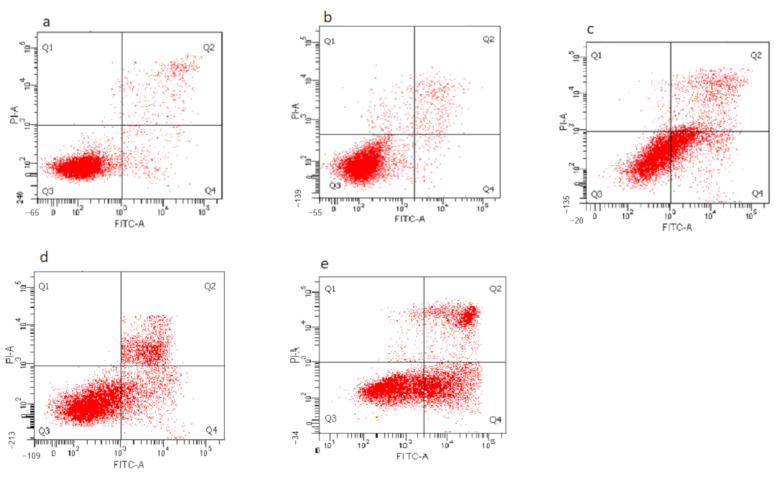
Dot plot of quadrant image of (Q1) necrotic cells, (Q2) late apoptotic cells, (Q3) viable cells, and (Q4) early apoptoic cells of the (**a**) untreated cell, (**b**) GOP nanocarrier, (**c**) PCA drug, (**d**) GOP–PCA nanocomposite and (**e**) GOP–PCA–FA nanocomposite.

**Figure 9:**
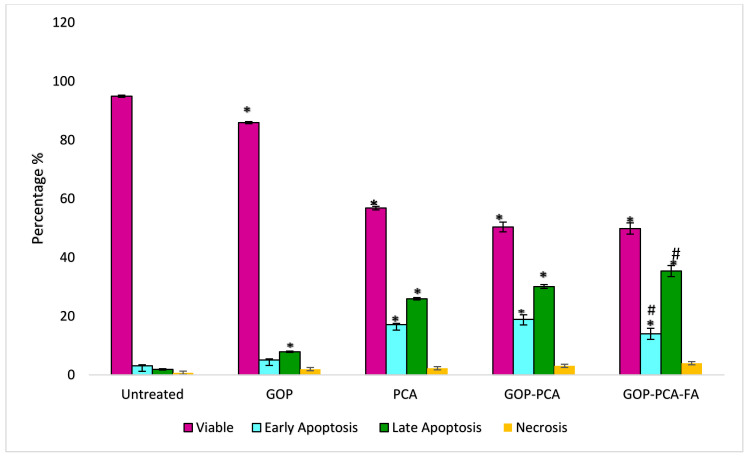
Histogram of quantitative analysis of viable, early apoptosis, late apoptosis and necrosis HepG2 cells. Cells were treated with GOP, PCA drug, GOP–PCA nanocomposite and GOP–PCA–FA nanocomposite at 38 μg/mL concentrations for 72 h. Values are expressed as mean ± SD of triplicates. The significant difference (*p* < 0.05) * was determined with untreated HepG2 cells against GOP, PCA, GOP–PCA and GOP–PCA–FA, and (*p* < 0.05) # using PCA treated against nanocomposites by one-way ANOVA followed by Games–Howell post hoc tests.

To determine the mode of cell death, HepG2 cells were incubated with the GOP, pristine PCA and nanocomposites followed by staining with Annexin V and PI. Figure 8 shows the plot dot quadrant image of viable, early apoptotic, late apoptotic and necrotic cells. In Figure 9 is shown a histogram depicting the percentages of untreated HepG2 cells at 93.89 ± 0.45% of viable cells, 3.58 ± 0.31% of early apoptotic cells, 1.89 ± 0.27% of late apoptotic cells and 0.73 ± 0.12% of necrotic cells. Meanwhile, HepG2 cells incubated with nanocarrier (GOP) showed the presence of 85.63 ± 0.47% viable cells, 5.11 ± 0.63% in early apoptosis, 7.29 ± 0.18% in late apoptosis and 1.97 ± 0.03% in the necrotic stage. The HepG2 cells treated with nanocarrier only exhibited significant changes at the late apoptosis stage compared to untreated HepG2 cells. When the cells were treated with pristine PCA, there were 55.80 ± 0.61% of the viable cells, with 42.05% in total, with both the early apoptotic and late apoptotic cells accounted for by the apoptosis, whereas the necrotic cells were only present at 2.27 ± 0.10%. The reduction in viable cells when treated with PCA was found to be significant (*p* < 0.05) compared to the untreated and GOP nanocarrier-traded. The increment in the percentage of apoptosis was significant compared to the control.

The percentages of viable cells were further reduced when cells were treated with GOP–PCA and GOP–PCA–FA nanocomposites. A significant reduction was seen in the viable cells compared to control—50.57 ± 1.66% and 41.85 ± 1.91%, respectively—while the percentage of apoptotic cell increased by 46.8% in the GOP–PCA nanocomposites and 55.36% in the GOP–PCA–FA nanocomposites, respectively. According to the comparison between the pristine PCA and nanocomposites, there was a significant (*p* < 0.05) reduction in GOP–PCA–FA nanocomposite-treated cells at the early apoptosis and late apoptosis stages. The overall data presented denote that the modes of cell death when treated with pristine PCA, GOP–PCA nanocomposite and GOP–PCA–FA nanocomposite in HepG2 cells were primarily apoptosis. 

### 3.9. The Effect of Nanocomposites on Cell Cycle Distribution in HepG2 Cells

**Figure 10:**
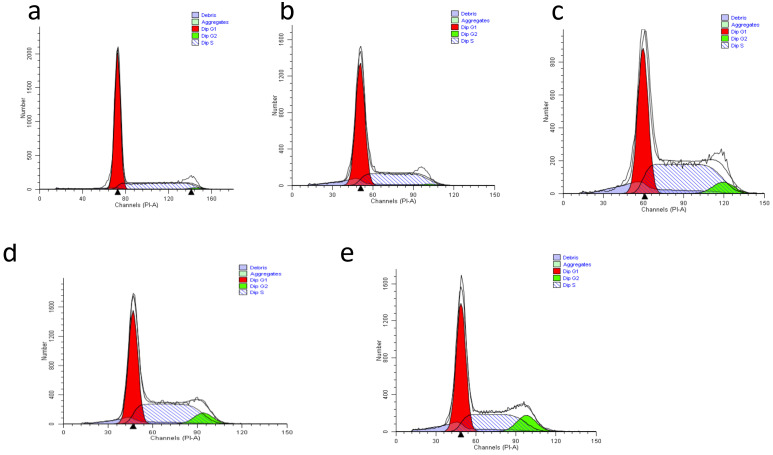
Representative cell cycle distribution of (**a**) untreated HepG2 cells, and those treated with (**b**) GOP nanocarrier, (**c**) PCA drug, (**d**) GOP–PCA nanocomposite and (**e**) GOP–PCA–FA nanocomposite.

**Figure 11:**
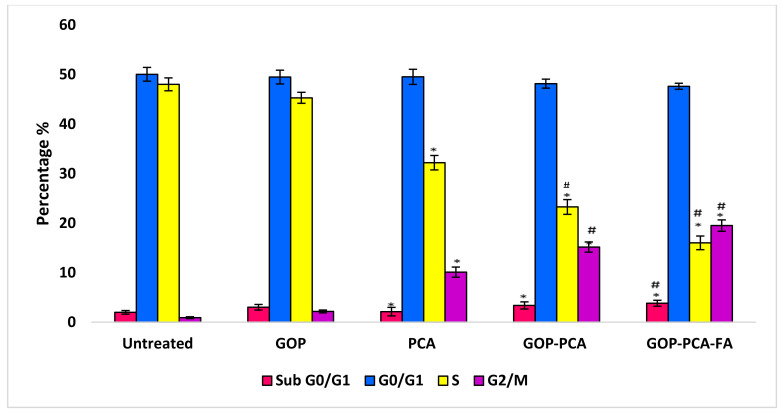
Histogram of quantitative analysis of cell cycle arrest (%) in HepG2 cells. Cells were treated with GOP, PCA, GOP–PCA and GOP–PCA–FA at 38 μg/mL concentrations for 72 h. Values are expressed as mean ± SD of triplicates. The significant difference (*p* < 0.05) * was determined between untreated HepG2 cells against GOP, PCA drug, GOP–PCA and GOP–PCA–FA nanocomposites, and (*p* < 0.05) # using the PCA drug treated against nanocomposites by one-way ANOVA followed by Games–Howell post hoc tests.

The protocatechuic acid treatment’s effects on the cell cycle in HepG2 cells were explored by flow cytometry analysis using PI staining. Figure 10 represents the cell cycle distribution of HepG2 cells treated with GOP, PCA, GOP–PCA and GOP–PCA–FA nanocomposites. Figure 11 represents a histogram of the percentages of cell cycle arrest at the sub G0/G1 phase, G0/G1 phase, S phase and G2/M phase. Meanwhile the untreated (control) cells exhibited almost similar G0/G1 and S phase distributions, of 50.27 ± 1.39% and 48.74 ± 1.30%, respectively. Treatment with GOP has an analogous pattern with untreated HepG2 cells, where a similar observation was seen in G0/G1 (51.51 ± 1.39%), but there was a significant reduction observed in the S phase, which was 5.42% from the untreated cells. When the HepG2 cells were treated with the PCA drug, GOP–PCA and GOP–PCA–FA nanocomposites, significant reductions in S phase were observed, whereby the percentages of cells were found to be 32.21 ± 1.46%, 23.27 ± 1.49% and 16.02 ± 1.38%, respectively. The accumulation of the G2/M phase was observed to be drastically increased in pristine PCA and nanocomposite-treated cells. Apart from that, cell accumulation in G2/M recorded increases of 10.11 ± 1.02%, 15.17 ± 1.03% and 19.51 ± 1.15%, respectively, compared to untreated cells. These results suggest that HepG2 cells treated with nanocomposites were arrested at the G2/M phase, with a parallel amplification in accumulation at the sub G0/G1 phase and decrease at the G0/G1 and S phases of the cell cycle.

### 3.10. Nanocomposite Stimulates Mitochondrial Membrane Potential in HepG2 Cells.

**Figure 12:**
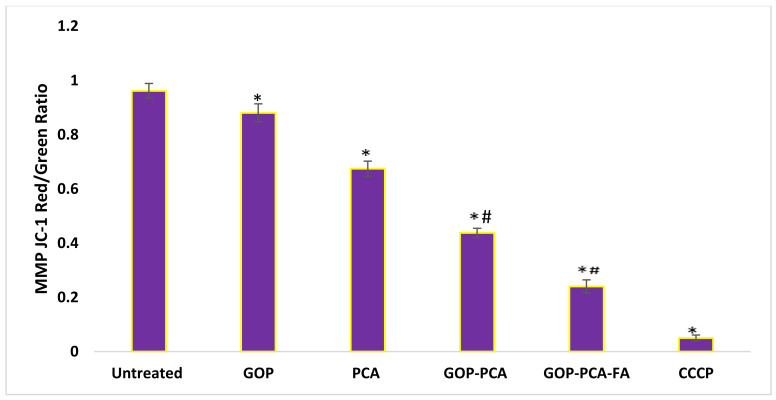
Mitochondrial membrane potential quantitative analysis for HepG2 cells. Cells were treated with GOP nanocarrier, PCA drug, GOP–PCA nanocomposite, and GOP–PCA–FA nanocomposite at 38 μg/mL concentrations for 72 h. Values are expressed as mean ± SD of triplicates. The significant differences (*p* < 0.05) * were determined between untreated HepG2 cells and GOP, PCA, GOP–PCA and GOP–PCA–FA nanocomposites, and (*p* < 0.05) # using PCA treated against nanocomposites by one-way ANOVA followed by Games–Howell post hoc tests.

The shift in mitochondrial membrane potential stimulated by protocatechuic acid was regulated using a fluorescent probe (JC-1). The ratio of red to green fluorescence shift in Δψm is displayed in Figure 12. The HepG2 cells that were incubated with DMEM medium predominantly exhibit red fluorescence, which indicates their composition of a 0.96 ± 0.027 ratio that can be translated as a high membrane potential. Contrarily, the incubation with GOP nanocarrier showed a significantly decreased to 0.88 ± 0.033 ratio, compared to the untreated cells. Treatment with PCA drug, GOP–PCA and GOP–PCA–FA nanocomposites caused a remarkable shift from reduction in red fluorescence to amplification of green fluorescence. When the HepG2 cells were treated with pristine PCA, the mitochondrial membrane potential decreased by 1.5-fold compared to untreated cells. A similar pattern was observed when the cells treated with GOP–PCA and GOP–PCA–FA nanocomposites exhibited decreases in 0.42 ± 0.017 and 0.24 ± 0.024 ratios, respectively. The HepG2 cells treated with GOP–PCA–FA nanocomposite showed most of their red aggregates shifting to green monomers. Following this, the significant differences (*p* < 0.05) were compared between the pristine PCA and nanocomposites, whereby a significant decrease in the ratio of mitochondrial membrane potential was shown, while CCCP was used as a positive control for this experiment. When the cells were treated with CCCP, the non-apoptotic cells presented a red fluorescence to green fluorescence ratio of 0.052 ± 0.011.

### 3.11. Nanocomposite Induces Intracellular ROS Generation in HepG2 Cells

**Figure 13:**
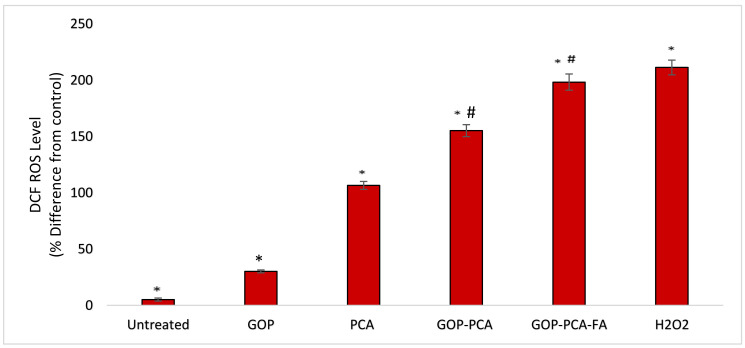
Reactive oxygen species (ROS) production in HepG2 cells for untreated cells and treated cell with GOP, PCA drug, GOP–PCA, GOP–PCA–FA nanocomposites and H_2_O_2_ at 38 μg/mL concentrations for 72 h. Values are expressed as mean ± SD of triplicates. The significant difference (*p* < 0.05) * was determined between untreated HepG2 cells and GOP, PCA, GOP–PCA and GOP–PCA–FA nanocomposites, and (*p* < 0.05) # using PCA treated against nanocomposites by one-way ANOVA followed by Games–Howell post hoc tests.

ROS plays an important role in facilitating cytotoxicity stimulated by chemotherapeutic agents. Figure 13 illustrates the effect of pristine PCA and nanocomposites on the production of intracellular ROS. Exposing HepG2 cells to H_2_O_2_ amplified the formation of ROS significantly, which served as a positive control for this study. HepG2 cells that were treated with GOP showed significant increments in the levels of ROS production as compared to untreated cells. Gradual increases in ROS generation were observed when cells were treated with pristine PCA, GOP–PCA nanocomposite and GOP–PCA–FA nanocomposite, respectively. Interestingly, in GOP–PCA and GOP–PCA–FA nanocomposites, the intracellular ROS generation in HepG2 was increased from 158.17 ± 3.91% to 190.27 ± 3.31%. This result indicates that there was a significant change in ROS generation between untreated and treated HepG2 cells. A comparison between the pristine PCA- and nanocomposite-treated cells exhibited a significant increase in the value of ROS generation.

### 3.12. Apoptosis-Related Proteins’ Expression in HepG2 Cells Treated with GOP–PCA–FA Nanocomposite

The molecular mechanisms accounting for GOP–PCA–FA nanocomposite-induced apoptosis in HepG2 cells were explored by detecting the expression levels of apoptosis-related proteins. Table 6 illustrates the quantitative analysis of the expression of apoptotic proteins in both untreated (control) and GOP–PCA–FA nanocomposite-treated HepG2 cells. These pro-apoptotic proteins were highly expressed by BAD, BAX, Pro caspase 3, cytochrome c, p21 and p53 following incubation with GOP–PCA–FA nanocomposite, whereas anti-apoptotic proteins, such as Bcl-2, Bcl-xL and HSP70, were shown to be significantly (*p* < 0.05) downregulated in treated HepG2 cells. 

### 3.13. Quantity Assessment Using qRT-PCR

The qRT-PCR gene expression levels of both untreated (control) HepG2 cells and GOP–PCA–FA-treated cells were quantitatively validated by Taqman real-time qRT-PCR. The expressions of targeted genes in the untreated and treated cells were normalized by the GAPDH house-keeping gene, where the fold change in the gene expression of each target gene was calculated and normalized by using the CFX Manager 3.0 Software (Bio-Rad) and efficiency-corrected method. 

**Figure 14:**
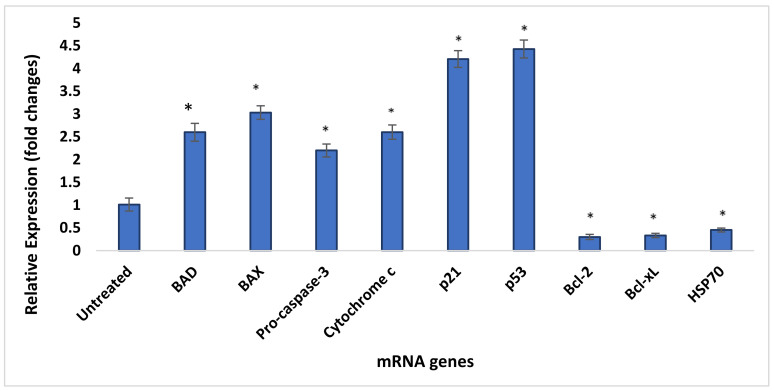
Effect of GOP–PCA–FA treatment on the expression of pro-apoptotic and anti-apoptotic genes’ expressions in HepG2 cells. HepG2 cells were treated with GOP–PCA–FA for 72 h, and the relative expression levels of pro-apoptotic and anti-apoptotic genes were analyzed by qRT-PCR. The results are expressed as mean ± standard deviation of triplicate. The treated cells showed statistically significant differences from the untreated cells by Student *t*-test.

Figure 14 shows the relative expression levels of pro-apoptotic and anti-apoptotic genes analyzed using qRT-PCR. There was significant upregulation of BAX, BAD, pro-caspase 3, cytochrome c, p53 and p21 genes in comparison with untreated control cells. The highest relative expression level was marked by the p53 gene’s expression, with a 4.4-fold change, followed by p21 with a 4.2-fold change. The rest of the genes’ expression changes were as follows: BAD (2.3-fold change), BAX (3-fold), Pro-caspase-3 (2.2-fold), and cytochrome c (2.6-fold). Similar to the protein microarray results shown earlier, the Bcl-2, Bcl-xL and HSP70 mRNA expressions were significantly downregulated, corresponding to the GOP–PCA–FA-treated cells when compared with the untreated (control) cells. The mRNA expression of Bcl-2 was 0.30-fold lower than the control, while Bcl-xL was 0.33-fold lower and HSP70 0.45-fold.

## 4. Discussion

Graphene oxide encompasses remarkable characteristic, such as a conjugated planar structure, ultrahigh surface area, exceptional mechanical and chemical stability, exceptional conductivity and proper biocompatibility [16]. Despite GO’s solubility in aqueous media, it has a charged screening effect that makes GO flakes aggregate [19]. The PEGylation process makes GO become more biocompatible and stable in all biological solutions [20]. This permits the delivery of anticancer drugs that bind to the graphene oxide surface through π–π stacking [21]. Conjugates such as GOP with high drug-loading are desirable, because they can contain higher amounts of drugs for massive uptake and transport to the targeted site [14]. The HPLC shows the percentage of drug-loading of the GOP–PCA nanocomposite to be 35.10%, and the GOP–PCA–FA nanocomposite was found to be 41.06%. The drug-loading percentage observed in our study was found to be comparable with previously reported work utilizing GO as a nanocarrier [14,22].

Active targeting on the cell surface receptors has been researched vigorously in cancer research, since many cancer cell types display upregulations of tumor-specific receptors [23]. One of the receptors that is overexpressed in multiple tumor cells is folate receptors (FR) (alpha subunit) [24,25]. The cellular uptake of FA is mediated in the mammalian cells by the folate receptor (FR) [26]. The folic acid-coated nanomaterial showed significant cellular uptake in the HepG2 cell [17,24,27]. The PEG-conjugated GO in this study enhanced the dispersibility of nanocomposites, thus providing flexibility for the interaction of FA ligands with multiple cell surface folate receptors that leads to improved specific uptake into cells [24].

The XRD analysis validated the crystallization and structure formation of the nanocarriers and nanocomposites. The XRD spectrum shows an increase in basal spacing from graphite to graphene oxide due to the addition of oxygenated functional groups such as carboxylic acid, hydroxyl and epoxides during the oxidation process [28], while the formation of a graphene oxide nanocomposite was also proven by the difference in peak formation during the addition of PEG polymer, free drug and folic acid. The disappearance of the peaks in the XRD spectra strongly suggests that there was a complete conversion of graphene oxide to graphene oxide nanocomposites [29]. The nanocomposites possessed a smaller particle size, which favors improving the bioavailability and enhancing the aqueous stability [30]. Smaller particle sizes and surface charges play a significant role in determining the protein adsorption and cellular interaction in physiological systems. The macrophages and phagocytes react strongly to the positively charged nanocomposite compared to those negatively charged [31]. In this case, the negatively charged nanocomposites have potential to increase the circulation half-life of the material by evading the immune system [32].

The release of bioactive compounds from the nanocomposites was evaluated in two different pH conditions, which were pH 4.8 (lysosomal) and 7.4 (blood). The burst release of PCA in the initial hours at pH 4.8 may be due to the dissociation of two possible H-bonds between the drug and the nanocarrier. These burst release phenomena are connected to the drugs that were attached to the nanocomposite surface via the partial dissociation of hydrogen bonding [26]. Cancer cells are rich in the intensification of new blood vessels that form from pre-existing vasculature, and the presence of cell surface receptors [25]. These blood vesicles and selected surface receptors are able to bring more nanocomposites to reach the targeted site and released bioactive compounds from nanocomposite due to the difference in the pH value [33]. As it is, the controlled drug release at pH 4.8 is considered the favorable condition for the anticancer treatment, due to the cancer environment, which is usually acidic [34].

We have reported the cell cytotoxicity by calculating the expression of intracellular lactate dehydrogenase enzyme (LDH) being released into the extracellular culture medium, which justifies the loss of cell integrity parallel to the gradual concentration increment. In a previous study, we have reported that the GOP nanocomposites have been uptaken by HepG2 cells via TEM image analysis^17^. This confirmed the penetration of GOP nanocomposites through the plasma membrane, and their internalization into the cytoplasm, mitochondria and nucleus [17]. This result also corresponds to the graphene oxide that has infiltrated into cells by piercing and mechanically interrupting the plasma membrane while gradually accumulating inside the cell compartment [17,35].

The clonogenic assay was performed to evaluate the inhibitory effect of the drug following the loss of reproductive integrity. The controlled release of the drug from nanocomposites is postulated to exhibit better antitumor efficacy due to the prolonged exposure of the anticancer drug to the cells [28]. As such, the clonogenic assay was conducted to evaluate the long-term efficacy of the prepared nanocomposites. The inhibition of clonogenic activity by GOP–PCA–FA was significantly higher compared to pristine PCA and the GOP–PCA nanocomposite in HepG2 cells. However, the nanocarrier system GOP has a minimal or no antagonistic effect on the clonogenic survival fraction, which indicates that the nanocarrier system was free from a cytotoxicity effect. This was further confirmed by Wang (2019) in a study conducted using ceramide–graphene oxide nanoparticles that showed no inhibitory effect on the proliferation of hepatocellular carcinoma cells when treated with nanoparticles [36].

In this study, flow cytometry was used to analyze the mode of cell death after the treatment of pristine PCA and nanocomposites by utilizing Annexin V-FITC/PI stains. The apoptotic cell heterogenous populations were divided and distinguished by FITC and PI cytofluorometric dye through differences in plasma membrane integrity and permeability [37]. During the apoptosis process, the phosphatidylserine (PS), which resides on the phospholipid membrane, was translocated, and the inner leaflet was exposed to the outer leaflet of the plasma membrane. This causes the phosphatidylserine to face the outer environment in the apoptotic cells [38]. The Annexin V binds to the exposed apoptotic cell surface PS at the early apoptosis stage. Phosphatidylserine translocation proceeds with the loss of membrane integrity. Here, the Annexin V bound to PS and PI is allowed to pass through the membranes and intercalate into nucleic acids. This is known as the late apoptosis stage [39].

Besides this, the folate-coated drug-loaded nanocomposite showed augmented apoptotic activity compared to its uncoated FA counterpart. The coating of FA on GOP–PCA efficiently increased the early and late apoptotic percentage in HepG2 cells. At the late apoptotic stage, the cell membrane starts to lose integrity, which was confirmed by the LDH release. Similar observations were made by Tian et al. (2016), wherein graphene oxide loaded with camptothecin drug and coated with folic acid showed efficient drug delivery and induced late apoptosis on HeLa cells, compared to the nanocomplex without folic acid [40]. In another study conducted by Yi et al. (2017), when doxorubicin (DOX), an anticancer drug, coated with folic acid on the graphene oxide (GOFA-DOX), was actively targeted to the MCF-7 breast cancer cell, showed a higher percentage of late apoptosis rate induced by the folic acid-coated nanocomposite compared to that without a folic acid coated or pristine doxorubicin [22].

The cell cycle mechanism ensures the proper replication of eukaryotic cells. In order for the cell to maintain balance, the cell growth and cell death processes have to be regulated and sustained. Multiple discoveries have supported that the changes in the expressions of cell cycle proteins may contribute to the promotion or inhibition of apoptosis [41]. Within cells, the DNA contents were evaluated by the affinity of PI stains to the DNA. The analysis shows the fluorescence intensity of single cells, differentiating them at the different phases of cell cycle arrest accordingly.

In this study, the HepG2 cell was arrested at the G2/M phase when treated with GOP–PCA and GOP–PCA–FA nanocomposites. The cell cycle arrested in the G2/M phase was proven to cause more permanent DNA damage [42]. The process of DNA damage was induced by anti-cancer drugs, such as camptothecin, doxorubicin, cisplatin, paclitaxel and 5-fluorouracil, which initiated p53-dependent and p53-independent pathways [43]. The G2/M checkpoint during cell cycle arrest hinders the cells from entering the mitosis phase [44,45]. Cell cycle arrest during the G2/M phase is considered to be a stimulating factor in cancer therapy, because tumor cells’ sensitivity is known to contribute to the DNA damaging effect of chemotherapeutic agents [46].

Reactive oxygen species (ROS) and oxidative stress are amongst the underlying mechanisms of nanomaterials’ toxicity [47]. ROS generation is known to be an important factor for the cell proliferation and differentiation mechanism [48]; however, it is suggested that excessive ROS generation may cause cell damage and eventually apoptosis [49]. In a study, the nanocomposites’ interaction with the cells led to excessive ROS generation through direct influence on the cell DNA or mitochondrial activity [35]. The mitochondrial permeability transition, which was led by ROS generation, resulted in the fluctuation of the outer mitochondrial membrane’s permeability, which channeled the cytochrome c release into the mitochondria. ROS is mainly produced in mitochondria [50]. As such, when mitochondrial ROS were released, this may have aggravated the damage to the cells. Graphene oxide nanocomposites induced the depolarization of mitochondrial membrane potential, which subsequently increased the generation of intracellular ROS. This mechanism eventually triggers apoptosis by activating the mitochondrial pathway [51]. In an observation between GO and carboxyl graphene nanoplatelets, the mitochondrial membrane has been indicated to cause an increase in intracellular ROS and plasma membrane damage-induced cytotoxicity in HepG2 cells [35].

The GOP–PCA–FA nanocomposite induces cell damage in HepG2 cells, causing the significant upregulation of BAX, BAD, caspase-3, cytochrome c, p21, and p53 pro-apoptotic proteins, whereas Bcl-2, Bcl-xL and HSP 70 proteins were found to be downregulated significantly. This study was further validated by qRT-PCR, which exhibited a similar pattern to the apoptotic microarray protein detection assay [52,53]. The cytochrome c that was upregulated during the outer mitochondrial membrane (OMM) permeabilization was considered to be highly apoptogenic, and has the capability to activate the caspase cascade mechanism [54]. Under normal conditions, the BAX proteins that usually exist abundantly in cell cytosol will assist in the transportation of the protein through the mitochondrial membrane by stimulating the transition pore permeability [55]. This will initiate the apoptosis mechanism by shifting the mitochondrial permeability [56]. When BAX, cytochrome c and caspase-3 are overexpressed, this may cause the mitochondria membrane to lose its permeability. This phenomenon is predicted to happen by our recent findings.

The cancer cells were postulated to survive by manipulating multiple mechanism in order to escape from apoptosis [57]. Other hypotheses could be derived when looking into the tumor immunology pathway, where the concurrent activation ofdifferent known regulated cell death pathways (RCD) as an alternative route to overcome cancer cell resistance phenomena. This should be looked into as an option for cell death in further studies [58,59,60]. The observations that we made clearly indicated that HepG2 cell death after nanocomposite treatment was associated with the upregulations of p53 and p21 gene expressions, assisted by the upregulations of BAD, BAX, cytochrome c and caspase-3 cascade reactions, which lead to cell death by the apoptosis mechanism. The p53 genes are well known as tumor suppressors [61]. They participate actively in the anticancer mechanism by activating the apoptosis mechanism via inducing genomic instability and inhibiting angiogenesis. Moreover, the tumor suppressing gene has been actively identified to be involved in cell cycle arrest [62], and in inducing programmed cell death during DNA damage [3]. The upregulation of genes p21 and p53 in HepG2 cells treated with nanocomposites notably increased with the fold changes of 4.2 and 4.4, respectively. This thought to be a plausible reason for the HepG2 cells being arrested at the G2/M phase. 

According to studies, cell cycle arrest at G2/M checkpoints functions in blocking the cell from entering into the mitosis phase during DNA damage or the cell undergoing stress^44,63^. The expression of the p21 protein is usually controlled by p53 protein [63]. As such, it is postulated that the expression of the p53 protein is regulated by the transition of the G2/M phase and the upregulation of the p21 protein, which could have inhibited cyclin B1–Cdc2 complexes in the cytoplasm, which is essential for cell entry into mitosis. Chung et al. (2017), reported that exposure to Sinularin for 24 h initiates the cell cycle arrest of the HepG2 cells at the G2/M phase, which is associated with the increased expression of p53 and p21 downstream proteins^3^. According to another study, the incorporation of gold–quercetin into poly(DL-lactide-co-glycolide) nanoparticles induces the apoptosis mechanism in HepG2 cells by activating p21, p53-ROS crosstalk and prompting epigenetic modifications. This event leads to inhibited proliferation together with the cell cycle arrest mechanism at the G2/M phase [49]. The GOP–PCA–FA nanocomposite not only forces HepG2 cells to undergo apoptosis, but triggers interconnected networks in the HepG2 cell death mechanisms. HepG2 cells undergo cell cycle mechanisms to be arrested at the G2/M phase when incubated with nanocomposites. These nanocomposites are capable of altering the mitochondrial membrane potential and triggering apoptosis through ROS generation. 

## 5. Conclusions

In conclusion, nanocomposite drug delivery demonstrated an improvement in cytotoxicity properties, which contributes to the significant anticancer mechanism of the GOP–PCA–FA nanocomposite compared to the GOP–PCA nanocomposite and pristine PCA in HepG2 cells. The comparison between GOP–PCA (passive targeting) and GOP–PCA–FA (active targeting) nanocomposites showed significant differences in terms of cell cytotoxicity in HepG2 cells. The upregulation of BAX, BAD, caspase 3, cytochrome c, p53, and p21 pro-apoptotic proteins, expressed through the treatment with the GOP–PCA–FA nanocomposite, not only forced HepG2 cells to undergo apoptosis, but triggered interconnected networks in the HepG2 cell death mechanisms. In a nutshell, the GOP–PCA–FA nanocomposite induced p53-mediated apoptosis by arresting the HepG2 cell cycle at the G2/M phase and depolarizing the mitochondrial potential while controlling the redox status of HepG2 cells. The formulation of the GOP–PCA–FA nanocomposite shows that the folic acid-coated active targeting system has resulted in a significant reduction in cell cytotoxicity, and increased the potential of anti-cancer treatment due to the controlled sustained release of PCA compared to the GOP–PCA nanocomposite and pristine PCA drug.

## Figures and Tables

**Table 1:** Reverse transcription polymerase chain reaction (RT-PCR) master mix components.

Component	Master Mix Volume/15 µL Reaction
100 mM dNTPs (with dTTP)	0.15
MultiScribe™ Reverse Transcriptase, 50 U/μL	1.00
10× Reverse Transcription Buffer	1.50
RNase Inhibitor, 20 U/μL	0.19
Nuclease-free water	4.16
Total	7.00

**Table 2:** Quantitative real-time PCR master mix components.

Component	Volume (µL)/20 µL Reaction
TaqMan gene expression Assay (20×)	1.00
Product from RT reaction (Minimum 1:15 Dilution)	1.33
TaqMan 2× Universal PCR Master Mix, No AmpErase UNGa	10.00
Nuclease-free water	7.67

**Table 3:** List of primers for RT-PCR.

Gene	Primer Sequences
Bad	**F**-5′-CAGGGGCCTCGTTATCGG-3′ **R**-5′-GGACTCTGGATCAGACCTCA-3′
Bax	**F**-5′-ATGTTTTCTGACGGCAACTTC-3′ **R**-5′-AGTCCAATGTCCAGCCCAT-3′
Caspase-3	**F**-5′-TGTTTGTGTGCTTCTGAGCC-3′ **R**-5′-CACGCCATGTCATCATCAAC-3′
Cytochrome c	**F**-5′-GCTACTCCTACCTATCTCCC-3′ **R**-5′-TGTGGTCGTTACCTAGAAGG-3′
p21	**F**-5′-TGGAGACTCTCAGGGTCGAAA-3′ **R**-5′-GGCGTTTGGAGTGGTAGAAATC-3′
p53	**F**-5′-ATGTTTTGCCAACTGGCCAAG-3′ **R**-5′-TGAGCAGCGCTCATGGTG-3’
Bcl-2	**F**-5′-ATGTGTGTGGAGACCGTCAA-3′ **R**-5′-GCCGTACAGTTCCACAAAGG-3′
Bcl-xL	**F**-5′-CAGAGCTTTGAACAGGTAG-3′ **R**-5′-GCTCTCGGGTGCTGTATTG-3′
HSP70	**F**-5′-AGGCCGACAAGAAGAAGGTGCT-3′ **R**-5′-TGGTACAGTCCGCTGATGATGG-3′
GAPDH	**F**-5′-GGCAAATTCAACGGCACAGT-3′ **R**-5′-AGATGGTGATGGGCTTCCC-3′

**Table 4:** Zeta potential were measured using Melvern Zetasizer.

Nanocomposites	ζ-Potential (mV)
GO	−29.6 ± 2.304
GOP	−9.92 ± 2.112
GOP–PCA	−15.5 ± 1.872
GOP–PCA–FA	−17.3 ± 2.007

**Table 5:** The amount of loading content and encapsulation efficiency of GOP–PCA and GOP–PCA–FA nanoparticles.

Nanocomposites	Loading Content (%)	Encapsulation Efficiency (%)
GOP–PCA	35.10%	97.03%
GOP–PCA–FA	41.06%	97.17%

**Table 6:** Quantitative analysis of proteins involved in apoptosis in GOP–PCA–FA-treated HepG2 cells at 19 μg/mL for 72 h.

Apoptotic Protein	Signal Intensity (Arbitrary Unit)		Fold Change
			(Treated/Untreated)
	Untreated	GOP–PCA–FA-Treated	
Pro-apoptotic proteins			
BAD	7823.15 ± 113.13	19,711.53 ± 154.16 *	2.53
BAX	7047.33 ± 102.91	19,790.15 ± 206.50 **	2.81
Pro-Caspase-3	8224.25 ± 193.75	15,682.80 ± 100.84 *	1.91
Cytochrome c	7177.67 ± 387.15	19,050.79 ± 394.31 **	2.62
p21	2682.75 ± 232.20	10,412.01 ± 136.61 ***	3.88
Phospho-p53 (S15)	2723.11 ± 269.43	10,851.03 ± 198.33 ***	3.99
Anti-apoptotic proteins			
Bcl-2	10,791.18 ± 241.87	2847.35 ± 183.76 ***	0.26
Bcl-xL	9645.18 ± 103.31	2833.87 ± 129.91 ***	0.29
HSP70	22,100.67 ± 175.72	9006.71 ± 414.04 **	0.41

Pro-apoptotic proteins’ and anti-apoptotic proteins’ expressions are indicated. Results expressed as mean ± SD, and * *p* < 0.05, ** *p* < 0.01, and *** *p* < 0.001 were considered significant by Student *t*-test.

## Data Availability

The data presented in this study are available on request from the corresponding author.

## Supplementary Materials

The following are available online at https://www.mdpi.com/1996-1944/14/4/817/s1, Figure S1: FTIR spectra of PCA, FA and GOP–PCA–FA nanocomposite, Table S1: FTIR bands of functional groups of free drug PCA, FA, GOP–PCA and GOP–PCA–FA nanocomposite, Figure S2: High-resolution transmission electron micrographs of (a) GO, (b) GOP, (c) GOP–PCACA and (d) GOP–PCACA-FA nanocomposite, Figure S3: Cell viability assay for normal human hepatocellular carcinoma (HepG2) cell line on protocatechuic acid and different functionalized graphene oxide nanocomposites; respectively, after 72 h of treatment, Table S2: IC50 value (μg/mL) of nanocarriers, pure compound and nanocomposites in different cell lines at 72 h of treatment.
