# Supplementary material for: Anticancer Molecular Mechanism of Protocatechuic Acid Loaded on Folate Coated Functionalized Graphene Oxide Nanocomposite Delivery System in Human Hepatocellular Carcinoma
Source: Materials (Basel). 2021 Feb 9;14(4):817. doi: 10.3390/ma14040817 (PMC7915244; doi:10.3390/ma14040817)
Supplement: Supplementary file 1 [file materials-14-00817-s001.pdf]

materials

MDPI

Article

Anticancer Molecular Mechanism of Protocatechuic Acid Loaded on Folate Coated Functionalized Graphene Oxide Nanocomposite Delivery System in Human Hepatocellular Carcinoma

Kalaivani Buskaran [1], Saifullah Bullo [2], Mohd Zobir Hussein [2], Mas Jaffri Masarudin [3], Mohamad Aris Mohd Moklas [4] and Sharida Fakurazi [1,4,*]

[1] Laboratory for Vaccine and Immunotherapeutic, Institute of Biosciences, Universiti Putra Malaysia, Serdang, Selangor 43400, Malaysia; vaneey_88@yahoo.com

[2] Materials Synthesis and Characterization Laboratory, Institute of Advanced Technology, Universiti Putra Malaysia, Serdang, Selangor 43400, Malaysia; bullosaif1@gmail.com (S.B.); mzobir@upm.edu.my (M.Z.H.)

[3] Department of cell and Molecular Biology, School of Biotechnology, Universiti Putra Malaysia, Serdang, Selangor 43400, Malaysia; masjaffri@upm.edu.my

4 Department of Human Anatomy, Faculty of Medicine and Health Sciences, Universiti Putra Malaysia, Serdang, Selangor 43400, Malaysia; aris@upm.edu.my

* Correspondence: sharida@upm.edu.my; Tel.: +603 9769 2352

Supplementary Materials

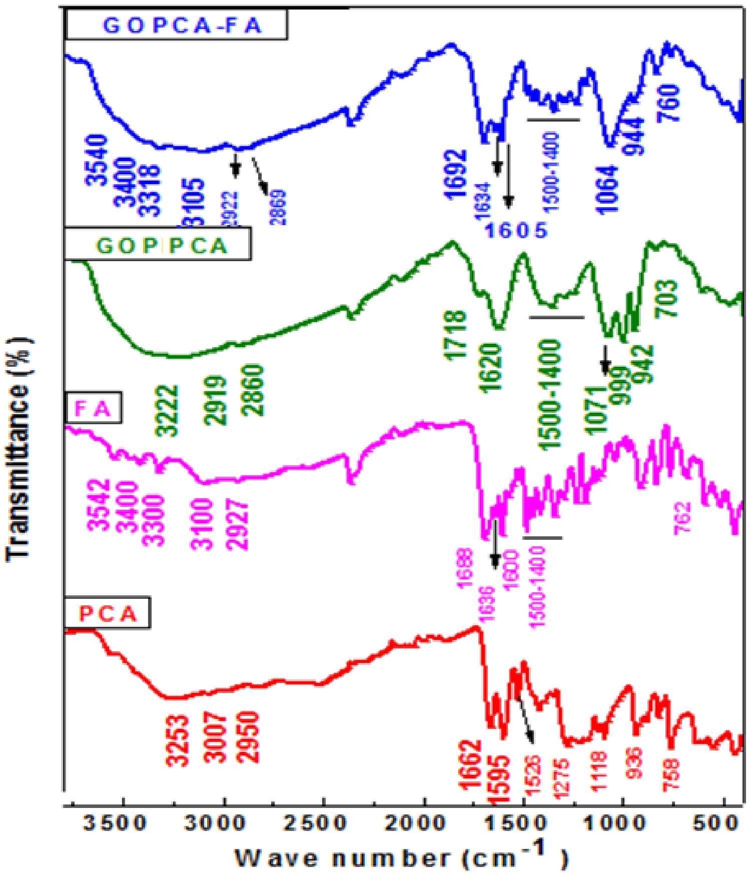

Figure S1. FTIR spectra of PCA, FA and GOP–PCA–FA nanocomposite.

Table S1. FTIR bands of functional groups of free drugs PCA, FA, GOP–PCA and GOP–PCA–FA nanocomposite.

Assignment	PCA	FA	GOP–PCA	GOP–PCA–FA
N-H	-	3542, 3460	-	3540, 3400
O-H stretching	3253	3319, 3414	3223	3318
C-H aromatic stretching and PEG stretching	3072	3091, 2927	2919, 2861	3110, 2922, 2869
C=O	1662	-	1620	1692
C=O amide	-	1688	-	1605
C=N	-	1636	-	1634
Aromatic C-C stretching	1500–1300	-	1500–1300	1500–1300
C-O-C ether	-	-	1071	1068
C-H in plane bending of aromatic ring	1040	-	1000	9043
C-H bending<br>N-H Rocking	760	762	703	761

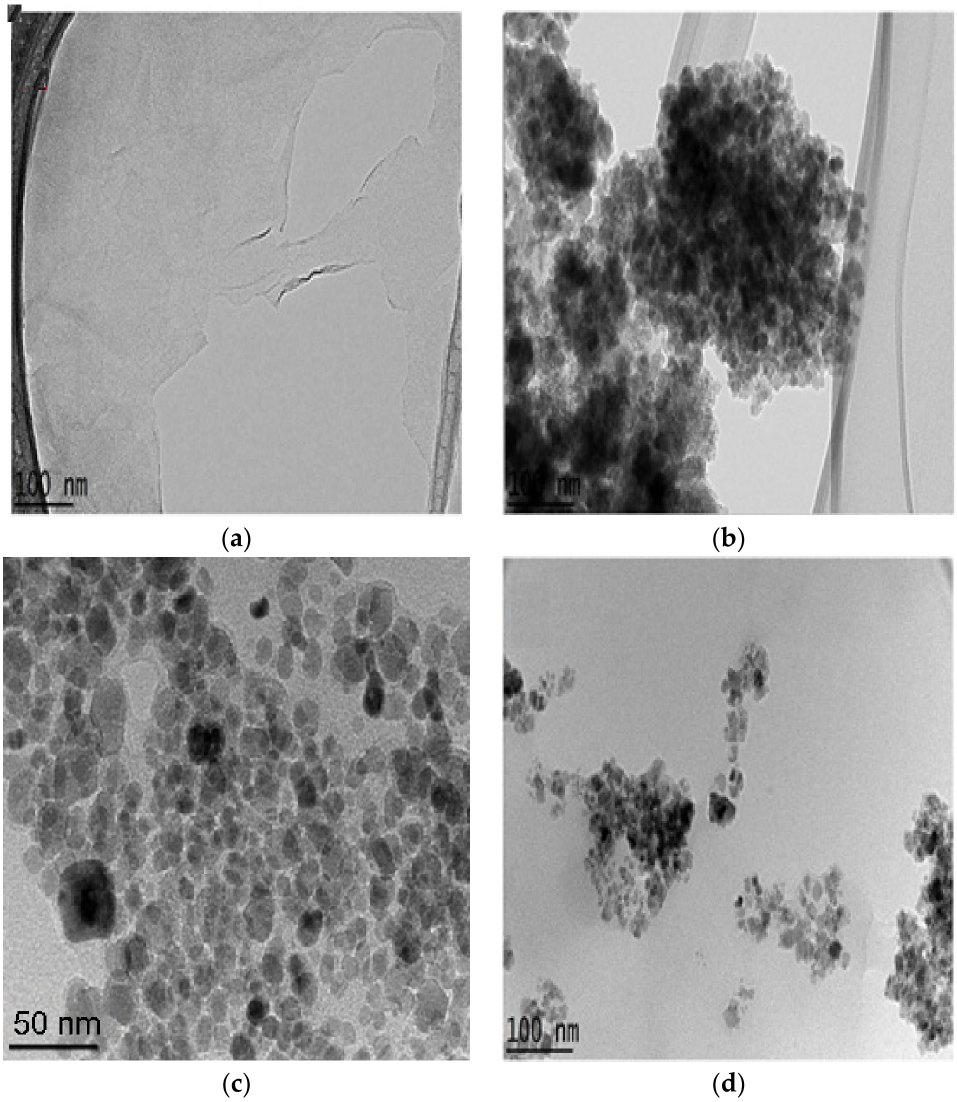

Figure S2. High-resolution transmission electron micrographs of (**a**) GO, (**b**) GOP, (**c**) GOP–PCACA and (**d**) GOP–PCACA–FA nanocomposite.

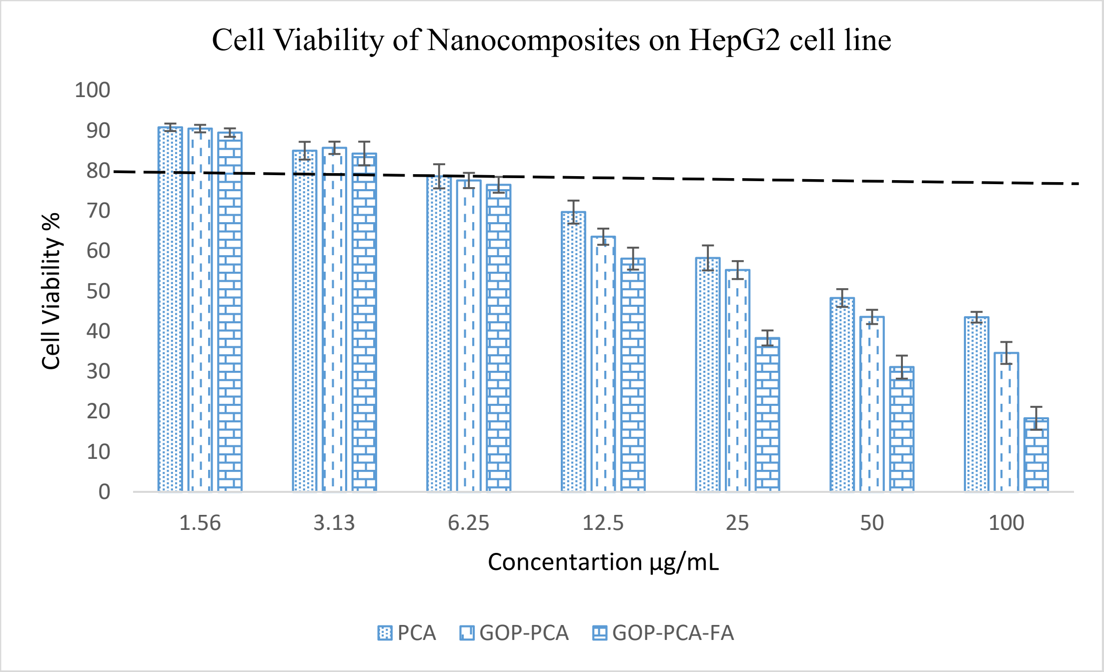

Figure S3. Cell viability assay for normal human hepatocellular carcinoma (HepG2) cell line on protocatechuic acid and different functionalized graphene oxide nanocomposites, respectively, after 72 h of treatment.

Results were calculated as mean ± standard deviation for n = 3 independent experiments. * $p < 0.05$ compared to PCA and CA (pure compound) treatment groups.

Table S2. IC_{50} value (µg/mL) of nanocarriers, pure compound and nanocomposites in different cell lines at 72 h of treatment.

CELL LINES	COMPOUND				
	GO	GOP	PCA	GOP–PCA	GOP–PCAFA
Hep G2	ND	ND	38.08 ± 2.22	*29.84 ± 2.78	*18.89 ± 1.64

The values are expressed as mean of three independent experiments. *(PCA) # (CA) P values < 0.05 were considered significant using one-way ANOVA followed by Dunnet's Post hoc test. (**ND: Not Determined**). IC_{50} derived from cell proliferation assay (MTT) showing the nanocomposite with higher anticancer potential on HepG2 and HT29 cells. Results represent the mean of three independent experiments.

Abbreviations: PCA, protocatechuic acid; CA, chlorogenic acid; GOP–PCACA–graphene oxide coated PEG and loaded with protocatechuic acid+ chlorogenic acid; GOP–PCACA–FA, graphene oxide-coated PEG and loaded with protocatechuic acid+ chlorogenic acid tagged with folic acid; GOP–PCA, graphene oxide-coated PEG and loaded with protocatechuic acid; GOP–PCA–FA, graphene oxide-coated PEG and loaded with protocatechuic acid tagged with folic acid.
